# Dynamic proton arc treatment planning study for oesophageal cancer

**DOI:** 10.1016/j.phro.2025.100837

**Published:** 2025-09-23

**Authors:** Macarena Chocan Vera, Anne-Catherine Wéra, Hamdiye Ozan, Erik Engwall, Viktor Wase, Otte Marthin, Johan Sundström, Sophie Wuyckens, Karin Haustermans, Ana M. Barragán-Montero, Kevin Souris, John A. Lee, Edmond Sterpin

**Affiliations:** a UCLouvain, Molecular Imaging, Radiotherapy and Oncology (MIRO), Brussels, Belgium; b Centre Hospitalier Universitaire CHU UCL Namur, Radiotherapy Department, Namur, Belgium; c RaySearch Laboratories, Stockholm, Sweden; d KULeuven, Department of Oncology, Laboratory of experimental radiotherapy, Leuven, Belgium; e Particle Therapy Interuniversity Center Leuven - PARTICLE, Leuven, Belgium; f Ion Beams Applications SA (IBA) Louvain-La-Neuve, Belgium

**Keywords:** Proton arc therapy, Proton therapy planning, Oesophagus, Robustness

## Abstract

Particle Arc Therapy (PAT) is considered a promising technique to improve conformity and reduce toxicities. Robustly optimized PAT plans were evaluated versus Intensity Modulated Proton Therapy (IMPT) for oesophageal cancer in 17 patients. Impact of motion, setup and range uncertainties on target coverage, plan quality and Organs At Risk (OAR) doses were assessed. PAT (two 80°–200°arcs) reduced OAR doses (spinal canal $D_{0.05{\text{cm}}^{3}}$: 5.12 Gy (12.8%), lungs and heart $D_{\text{mean}}$: 0.39 Gy (8.8%) and 0.83 Gy (10.5%)) while maintaining robustness. Similar toxicities were observed, but delivery time was doubled for PAT, indicating that further development is needed.

## Highlights

• Dynamic arc improved organ sparing.
• Delivery time was doubled versus conventional protons.

## Graphical abstract

**Figure:**
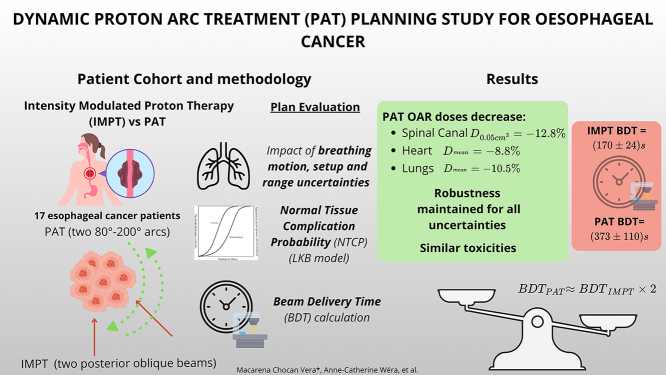

## 1 Introduction

The standard of care for locally advanced oesophageal cancer patients is a trimodal treatment involving chemoradiotherapy (CRT) as induction therapy before proceeding to surgical resection or definitive CRT if resection is not possible [1], [2]. The proximity of the oesophagus to organs at risk (OARs) like the heart, lungs and spinal canal emphasizes the need of dose conformation to limit toxicities. Besides dose-limiting oesophageal toxicities, pulmonary and cardiac complications are often reported [3], [4], [5].

Intensity modulated proton therapy (IMPT) has proven to enhance conformality and OAR sparing [6], [7], potentially leading to reduced heart and lung toxicities [8]. Additional conformity and OAR sparing could be achieved by making use of more degrees of freedom in the plan.

Dynamic Proton Arc Therapy (PAT) is a promising approach [9], [10], [11], [12] that could reduce the impact of breathing motion and mitigate the impact of uncertainties in thoracic tumours [13], [14], [15]. It could improve target dose conformity and lower dose to OARs leading to reduced toxicities while maintaining tumour coverage [14], [15], [16], [17], [18], [19].

Regarding dynamic PAT clinical application, the main challenge in plan optimization algorithms is the much higher number of degrees of freedom to explore. Since the gantry rotates continuously around the patient, the number of beam incidences and energy layers (EL) in the plan increases, hence the need for a fast and efficient energy selection algorithm. Recently, substantial efforts have been made to develop delivery-efficient PAT plans [20], [21], [22], [23], [24], [25]. This has culminated in the implementation of methods that rely on pre-selecting energy layers and placing the spots before running the optimization, like the ELSA algorithm (Early Energy Layer and Spot Assignment) [26].

While we previously evaluated ELSA algorithm for lung cancer [13], this work focuses on oesophageal cancers. To do so, IMPT and PAT plans are compared in terms of plan quality and robustness against setup/range errors, breathing motion, beam delivery time (BDT) and Normal Tissue Complication Probability (NTCP), with the goal of assessing potential benefits of ELSA-based PAT for this tumour location.

## 2 Materials and methods

### 2.1 Patient database and treatment planning

The database consisted of 17 patients with locally advanced oesophageal cancer. The use of patient data for the study was approved by the Institutional Ethical Review Board of UZLeuven (S59667). This work complies with the protocol of the PROTECT clinical trial (NCT05055648) [27].

Each patient was scanned with 4D computed tomography (4DCT). Respiratory motion was accounted for with an iCTV, i.e. the sum of the CTV in all phases of the 4DCT. The iCTV density was overridden to be equal to muscle (1.05 g/cm${}^{3}$) to account for sudden density changes due to target movement.

All patients were planned with a biological-weighted dose prescription of 50.4 Gy with an RBE of 1.1 [28] delivered in 28 fractions. Treatment plans were generated with RayStation 11B (RaySearch Laboratories, Sweden) for IMPT and a research version of RayStation 12 A for PAT, for an IBA ProteusPlus machine. All plans were optimized using the Monte Carlo dose engine (v5.3) embedded in RayStation, for both beamlet (10,000 ions/spot) and final dose calculation (0.5% uncertainty). No range shifters were required. More information can be found in Supplementary material A.

Robustness evaluation was performed on the planning CT (average CT) using 7 mm isotropic patient setup error combined with range error (0% and ±2.6%), resulting in a total of 42 scenarios for each delivery technique [29], [30].

To evaluate the impact of motion, the nominal scenario was recomputed in each breathing phase composing the 4DCT (between 8 and 10 available phases). In that case, the evaluated target is the CTV.

The clinical goals for the nominal case are reported in Table S1. Milder criteria were used for robustness evaluation on setup scenarios and breathing phases.

### 2.2 Plan evaluation and comparison

For each patient and plan, dose-volume metrics associated with the clinical goals reported in Table S1 were evaluated.

Target dose homogeneity (Homogeneity Index (HI) [31]), target conformity (Conformity Index (CI) [32]) and body integral dose (ID), i.e. the product of mean body dose and patient volume in cm${}^{3}$ were evaluated.

Lung and heart toxicities for both modalities were calculated using the Lyman-Kutcher-Birman NTCP model [33], based on mean doses (mean heart dose: MHD and mean lung dose: MLD). More details on the calculation can be found in Supplementary material B (formulas S1–S4).

Statistical analyses comparing target and OARs metric results between the two delivery techniques were conducted using a Wilcoxon matched-pairs test with the Python SciPy library. Median differences were calculated for each metric, along with their respective interquartile ranges (IQR).

### 2.3 Beam delivery time

For PAT plans, beam delivery time (BDT) was estimated using the arc trajectory optimization method (ATOM) [34]. All parameters used for BDT calculation can be found in Table S2. For IMPT, a simple start-and-stop trajectory was calculated. While human intervention time during IMPT beam delivery was not considered, travel time between beams, including couch and gantry rotation, was accounted for. To this date, no clinically approved model to calculate beam delivery time for arc trajectories has been published.

## 3 Results

All median differences and their respective IQR are reported in Table S3. In the nominal case, there was no clinically relevant difference in target coverage ($D_{98\text{\%}}$, $D_{95\text{\%}}$, $D_{2\text{\%}}$) across techniques but PAT yielded statistically significant better-quality indices than IMPT (Table S3). All clinical goals for the target were achieved. An example of dose distribution and dose map difference is shown in Figure S1.

In the worst case scenario for setup/range uncertainties, there were no statistical difference in dose-volume metrics. PAT yielded a smaller $D_{2\text{\%}}$ (IMPT $D_{2\text{\%}}$ = 52.07 Gy vs PAT $D_{2\text{\%}}$ = 51.41 Gy), which could imply a better chance of avoiding high doses inside the target.

When comparing $V_{95\text{\%}}$ for the plan recalculation on each breathing motion phase (Table S3), there was a small but not clinically important difference in favour of IMPT, with both techniques achieving $V_{95\text{\%}}$ >99%.

**Fig. 1:**
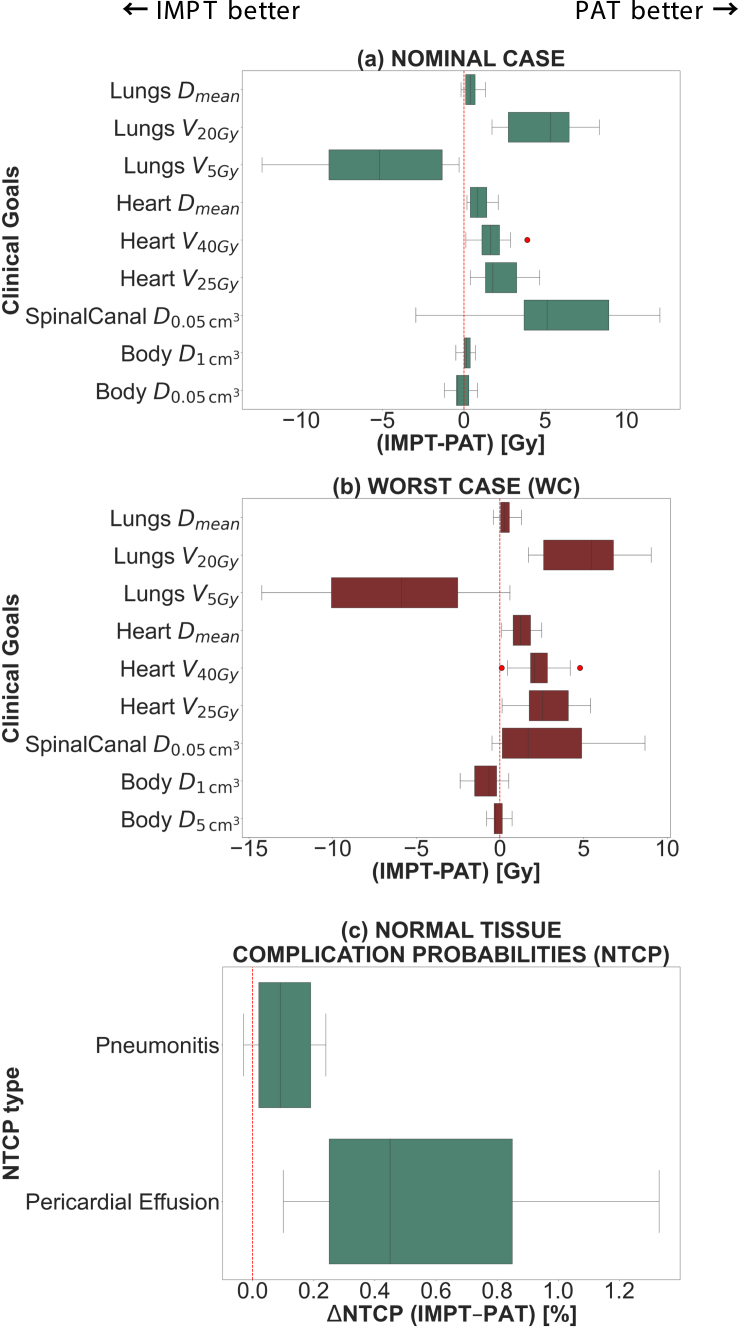
Plan comparison for the OARs, considering all scenarios. Median differences and their respective deviations from the median value were computed for all metrics. Outliers are represented by red dots. Interquartile range (IQR, Q1–Q3) shown in boxes; Q1 = 25th percentile, Q3 = 75th percentile (a) Nominal case (b) Worst case scenario for setup and range uncertainties (c) NTCP values for two toxicity types, pneumonitis and pericardial effusion, namely.

For the nominal case, relevant OAR metrics are shown in Fig. 1a. For PAT, as shown by the p-value and IQR presented in Table S3, statistically significant differences were found for all structures decreasing max dose delivered to spinal canal (−12.8%), MLD and lungs $V_{20Gy}$ (−8.8% and −47.8%, respectively), as well as MHD (−10.5%), heart $V_{40Gy}$ (−19.9%) and $V_{25Gy}$ (−13.1%). The reduction in lungs $V_{20Gy}$ came at the expense of increasing $V_{5Gy}$ by 29.6% for PAT. On the other hand, this technique presented a slight decrease in body integral dose by 1.8%.

In the worst case scenario, PAT plans were less sensitive against uncertainties (Fig. 1b-Table S3), preserving MLD, MHD and max dose to the spinal canal. On the contrary, PAT presented a minimal plan robustness deterioration regarding max body dose, resulting in higher $D_{1\;\text{cm}{}^{3}}$.

The impact of breathing motion was also evaluated on relevant structures (Table S3). Although for both treatment modalities, the clinical goal for the spinal canal was always below the threshold of 50 Gy in all phases, PAT managed to reduce the $D_{1\;\text{cm}{}^{3}}$ by 10.8%, on average. However, PAT was not as effective when handling hot spots in the body, with IMPT showing a slightly lower but statistically significant $D_{1\;\text{cm}{}^{3}}$.

The small differences in MLD and MHD in the nominal case translated into minor deviations when assessing NTCP associated with pulmonary and cardiac complications (Table S4). The results, illustrated in Fig. 1c, showed that PAT reduced NTCP values to a very small extent (<1%).

**Fig. 2:**
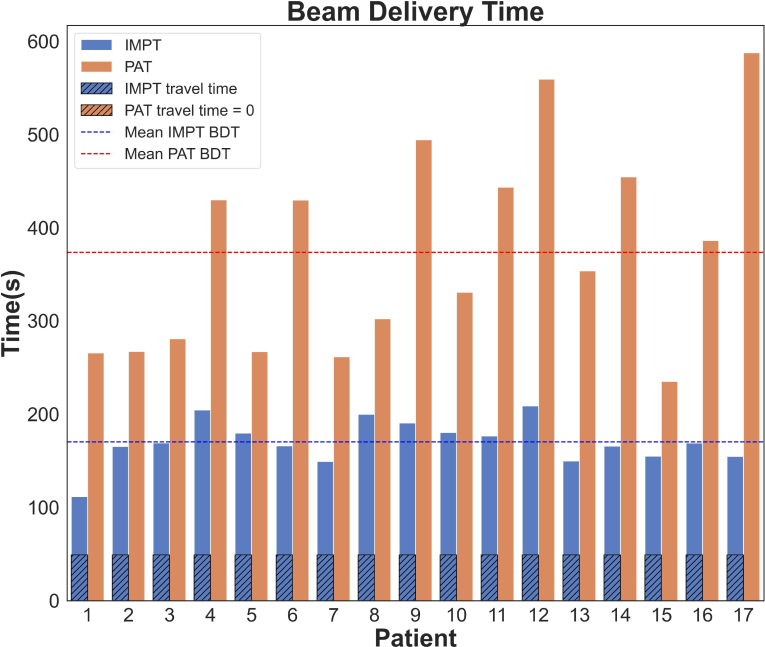
Beam Delivery Time (BDT) for IMPT (blue bars) and PAT (orange bars) for 17 oesophageal cancer patients. IMPT lagging time in between beams is shown as a dashed overlapped bar over the IMPT BDT representation (49 s for all patients). Mean BDT is represented by the dashed line.

BDT results for both modalities and all patients are reported in Fig. 2 showing a significant difference of 54% longer BDT for PAT. The average BDT for the 2-beam IMPT plans, including 49 s for gantry rotation, was (170±24 s), while for PAT plans, with 2 arcs and either 1 or 2 revolutions per arc, BDT was (373±110 s). Moreover, as shown in Fig. 2, PAT was slower than IMPT for each patient individually.

## 4 Discussion

In this study on oesophageal cancer, ELSA-based PAT improved dose homogeneity and reduced OAR doses (spinal canal, lungs, heart) in comparison to IMPT, but doubled the beam delivery time. Differences in robustness between PAT and IMPT were clinically insignificant when accounting for setup, range errors, and breathing motion.

Those results are in agreement with our prior research on PAT for lung target [13], ELSA-based PAT improved dose conformity and plan quality but showed reduced robustness under non-gated breathing conditions. The current posterior 2-beams configuration in IMPT balances tissue sparing and robustness effectively and we applied similar beam angles (80°–200°) in PAT. Expanding to a full 360°arc could improve plan quality but risks diaphragm-related issues.

Beside the ability of PAT to reduced dose to OARs, there could be other benefits of PAT planning, such as optimizing linear energy transfer (LET) distribution. Indeed, with PAT, the edge of the tumour would not need to be covered using the distal edge of the spread-out Bragg peak. Thus, high LET could be refocused to the centre of the target, increasing the relative biological effectiveness within tumour while decreasing it in OARs [22].

Among the advantages of PAT, is its presumed ability to reduce beam delivery time [17], [35] and, therefore, total treatment time as, unlike IMPT, there is no delay in between successive beam angles. However, since BDT calculation relies strongly on machine parameters, like energy layer switching up/down time, time per spot switch, spot delivery time per MU, total amount of energy layers/spots in the plan, and arc beam configuration chosen, this may be situation specific. In our recent study on lung cancer [13], on average, the BDT was similar for our 3 beam IMPT plans and single-arc PAT plans. From our calculations, and relying only on estimated BDT with ATOM model, the studied PAT plans for oesophageal tumours would take on average more than twice as long to deliver than the IMPT plan. However, this result is highly dependent on initial settings like the number of arcs and total EL/spots (Table S5). Notice that while for IMPT, 2 posterior beams and 67 EL total on average were enough to achieve excellent plan quality and robustness against all uncertainties, PAT required multiple arcs to achieve similar results leading to a 60% increase in ELs.

Although increasing the lung low-dose volume, a single-revolution full-arc could be a better option to preserve the overall plan quality and reduce the total number of layers and spots leading to reduced BDT closer to IMPT values. Algorithmically, BDT could be included in the objective function as proposed by Wuyckens et al. [24]. Finally, in terms of machine hardware, further decreasing EL switching time will contribute to further shortening of the BDT [14], [17], as currently it can take up to 70% of the total BDT for regular proton-therapy treatments [36], [37]. Nevertheless, the total treatment time could potentially still be shorter for PAT compared to IMPT treatment thanks to its simplified workflow.

Significant technical development is required for a wide clinical deployment of PAT [12] but current efforts also target software development, with optimization and planning algorithms. The ELSA algorithm offers a good trade-off between optimization time and plan quality due to early energy layer selection. Many other energy pre-selection and spot filtering algorithms have been proposed with the goal of shrinking the optimization problem [20], [25], [38], [39]. However, these methodologies might also cause a decrease in the degrees of freedom available for PAT preventing to realize the full potential of the technique. One solution for this problem can be to select EL based on the geometric coverage of the tumour penalized as a function of the crossing of selected organs at risk [40].

## CRediT authorship contribution statement

**Macarena Chocan Vera:** Conceptualization, Methodology, Validation, Formal analysis, Investigation, Data curation, Writing – original draft, Visualization. **Anne-Catherine Wéra:** Conceptualization, Methodology, Formal analysis, Investigation, Data curation, Writing – review & editing, Visualization. **Hamdiye Ozan:** Methodology, Validation. **Erik Engwall:** Software, Writing – review & editing. **Viktor Wase:** Software. **Otte Marthin:** Software. **Johan Sundström:** Software. **Sophie Wuyckens:** Software, Visualisation. **Karin Haustermans:** Resources. **Ana M. Barragán-Montero:** Supervision, Project administration, Writing – review & editing. **Kevin Souris:** Supervision, Project administration. **John A. Lee:** Project administration, Funding acquisition. **Edmond Sterpin:** Supervision, Project administration, Funding acquisition, Writing – review & editing.

## Declaration of competing interest

The authors declare the following financial interests/personal relationships which may be considered as potential competing interests: Macarena Chocan Vera, Anne-Catherine Wera, Hamdiye Ozan and Sophie Wuyckens benefited from a financial support of Wallonia in the frame of a MecaTech’s and BioWin’s Clusters program. John A. Lee is a Research Director with the Belgian F.R.S.-FNRS. Erik Engwall, Viktor Wase, Otte Marthin, Johan Sundström are employees of RaySearch Laboratories AB.

## Appendix A Supplementary data

MMC S1 Supplementary material includes patient database, planning details, NTCP model, and additional result data.
