# Supplementary material for: Dynamic proton arc treatment planning study for oesophageal cancer
Source: Phys Imaging Radiat Oncol. 2025 Sep 23;36:100837. doi: 10.1016/j.phro.2025.100837 (PMC12517070; doi:10.1016/j.phro.2025.100837)
Supplement: MMC S1 — Supplementary material includes patient database, planning details, NTCP model, and additional result data. [file mmc1.pdf]

Supplemental Material

A Patient database and treatment planning

Following PROTECT guidelines, IMPT plans were made using two oblique posterior beams (150° and 180°) on the average CT with a calculation grid size of 2.5x2.5x2.5 mm^3. Worst-case minimax robust optimization was performed on the iCTV target and the spinal canal dose-volume objectives with 7 mm setup error [1] and 2.6% range error [2]. For PAT, the dedicated optimization algorithm ELSA [3] was used with two entangled arcs (80°-200°) in opposing rotation with 2° angular spacing. When the target could not be properly covered, two revolutions per arc were generated. The plans were robustly optimized with the same conditions as IMPT except for the spinal canal, as it was not necessary. This limited arc range was chosen to limit dose deposition to the heart and contralateral lung, as well as to try to mitigate the effect of breathing motion.

B Lyman-Kutcher-Birman NTCP model

IMPT and PAT plans obtained for each patient are compared in terms of NTCP. For this purpose, we chose the LKB (Lyman-Kutcher-Birman) NTCP model [4]. The model is defined by:

$$NTCP = \frac{1}{\sqrt{2\pi}} \int_{-\infty}^{t} e^{-\frac{x^2}{2}}\, dx \tag{S1}$$

$$t = \frac{D_{\text{eff}} - TD_{50}}{mTD_{50}} \tag{S2}$$

$$D_{\text{eff}} = \left(\sum_i (v_i D_i)^{\frac{1}{n}} \right)^{n} \tag{S3}$$

D_{eff} is the dose that, if given uniformly to the entire volume, will lead to the same NTCP as the actual non-uniform dose distribution. TD_{50} is the uniform dose given to the entire organ that results in 50% complication risk. m is a measure of the slope of the sigmoid curve, n is the volume effect parameter (if n=1, D_{eff} corresponds to mean dose), V_{i} is the fractional volume receiving D_{i}. In this work, with 1.8 Gy per fraction, D_{i} will be replaced by $LQED2_{\text{i}}$:

$$LQED2_{\text{i}} = D_{\text{i}} \frac{1 + \frac{\frac{D_{\text{i}}}{N}}{\frac{\alpha}{\beta}}}{1 + \frac{2}{\frac{\alpha}{\beta}}} \tag{S4}$$

Where N is the number of fractions. $LQED2_{\text{i}}$ converts D_{i} into isoeffective $2Gy$ fractions. The parameters TD_{50}, n, m and $\frac{\alpha}{\beta}$ are tissue and complication specific and are found in the literature. More details can be found in [4].

When radiation pneumonitis is considered for the lungs, from [5], the parameters used for the NTCP calculation are: D_{eff} = MLD ($n = 1$) , TD_{50} = 29.9 Gy, $m = 0.41$.

Regarding heart toxicity, from [6], the parameters used for pericardial effusion probability are: D_{eff} = MHD ($n = 1$) , TD_{50} = 34.3 Gy, $m = 0.75$.

Table S1: Clinical Goals for target (iCTV in the nominal and setup/range scenarios, CTV for breathing motion scenario) and relevant OARs. MLD= Mean Lung Dose, MHD= Mean Heart Dose

	Nominal case	Scenarios (Worst Case)	Breathing Motion (Worst Case)
iCTV	$D_{98\%} \geq 97\%$<br>$D_{95\%} \geq 95\%$	$D_{98\%} \geq 95\%$<br>$D_{95\%} \geq 95\%$	
CTV			$V_{95\%} \geq 97\%$
Spinal Canal	$D_{0.05\mathrm{cm}^3} \leq 45\,\mathrm{Gy}$	$D_{0.05\mathrm{cm}^3} \leq 50\,\mathrm{Gy}$	
Body	$D_{0.05\mathrm{cm}^3} \leq 110\%$<br>$D_{1\mathrm{cm}^3} \leq 107\%$	$D_{1\mathrm{cm}^3} \leq 110\%$<br>$D_{5\mathrm{cm}^3} \leq 107\%$	
Lungs	MLD $\leq 20\,\mathrm{Gy}$<br>$V_{20\,\mathrm{Gy}} \leq 35\%$<br>$V_{5\,\mathrm{Gy}} \leq 70\%$		
Heart	MHD $\leq 26\,\mathrm{Gy}$<br>$V_{40\,\mathrm{Gy}} \leq 30\%$<br>$V_{25\,\mathrm{Gy}} \leq 50\%$		

Table S2: ProteusPlus Machine parameters.

Energy Layer Switching time up [s]	6.0
Energy Layer Switching time down [s]	0.8
Gantry max velocity [deg/s]	6.0
Gantry max acceleration s^2	0.6
Time per spot switch [ms]	2
Spot delivery time per MU [ms/MU]	5
Dead time per energy layer [s]	0.3

Table S3: Results for targets and OARs. The metrics for iCTV are evaluated only in the nominal and worst-case scenario of setup and range uncertainties, while only $V_{95\%}$ for the CTV is evaluated in the worst-case scenario for breathing motion across all phases. Median Δ values for OARs are shown; Δ = IMPT − PAT > 0 means PAT is superior for that metric. N/E = not evaluated, WC = worst-case, ID = integral dose, IQR = Interquartile Ranges, IMPT = Intensity Modulated Proton Therapy, PAT = Particle Arc Therapy.

	Clinical Goal	Nominal		WC setup/range		WC breathing motion	
		Δ (IQR)	p-val	Δ (IQR)	p-val	Δ (IQR)	p-val
**Target**							
iCTV	$D_{98\%}$ [Gy]	$-0.3(-0.4- -0.2)$	$\ll 0.001$	0.1(0.07–0.5)	0.01	N/E	N/E
	$D_{95\%}$ [Gy]	$-0.3(-0.30- -0.2)$	$\ll 0.001$	$0.02(-0.1–0.2)$	0.5	N/E	N/E
	$D_{2\%}$ [Gy]	0.3(0.2–0.4)	$\ll 0.001$	$0.08(-0.09–0.2)$	0.4	N/E	N/E
	CI	0.2(0.1–0.3)	$\ll 0.001$	N/E	N/E	N/E	N/E
	HI	0.01(0.01–0.01)	$\ll 0.001$	N/E	N/E	N/E	N/E
CTV	$V_{95\%}$ [%]	N/E	N/E	N/E	N/E	0.2(0.1–0.4)	$\ll 0.001$
**OARs**							
Lungs	D_{mean} [Gy]	0.4 (0.1 – 0.7)	$\ll 0.001$	0.3 (0.04 – 0.6)	0.01	N/E	N/E
	$V_{20\mathrm{Gy}}$ [%]	5.3 (2.7 – 6.5)	$\ll 0.001$	5.5 (2.6 – 6.8)	$\ll 0.001$	N/E	N/E
	$V_{5\mathrm{Gy}}$ [%]	−5.2 (-8.3 – -1.3)	$\ll 0.001$	$-5.9(-10.1- -2.5)$	$\ll 0.001$	N/E	N/E
Heart	D_{mean} [Gy]	0.8 (0.4 – 1.4)	$\ll 0.001$	1.3 (0.8 – 1.8)	$\ll 0.001$	N/E	N/E
	$V_{40\mathrm{Gy}}$ [%]	1.6 (1.1 – 2.2)	$\ll 0.001$	2.1 (1.8 – 2.9)	$\ll 0.001$	N/E	N/E
	$V_{25\mathrm{Gy}}$ [%]	1.8 (1.3 – 3.3)	$\ll 0.001$	2.6 (1.8 – 4.1)	$\ll 0.001$	N/E	N/E
Spinal Cord	$D_{0.05\mathrm{cm}^3}$ [Gy]	5.1 (3.7 – 8.9)	$\ll 0.001$	1.7 (0.1 – 4.9)	0.01	4.3 (3.1 – 8.5)	$\ll 0.001$
Body	$D_{0.05\mathrm{cm}^3}$ [Gy]	−0.4 (-0.5 – 0.3)	0.2	N/E	N/E	N/E	N/E
	$D_{1\mathrm{cm}^3}$ [Gy]	0.2 (0.02 – 0.4)	0.06	$-0.7(-1.5- -0.2)$	0.001	$-0.6(-0.9- -0.2)$	$\ll 0.01$
	$D_{5\mathrm{cm}^3}$ [Gy]	N/E	N/E	$-0.3(-0.4–0.1)$	0.11	$-0.2(-0.4- -0.1)$	N/E
	ID [Gy·L]	2.5 (-1.7 – 6.4)	0.1	N/E	N/E	N/E	N/E

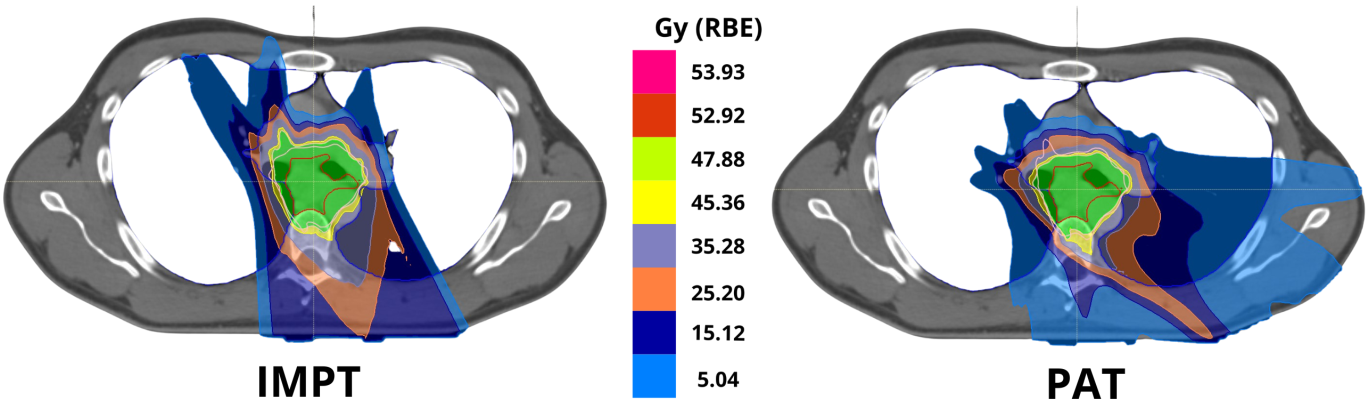

(a)

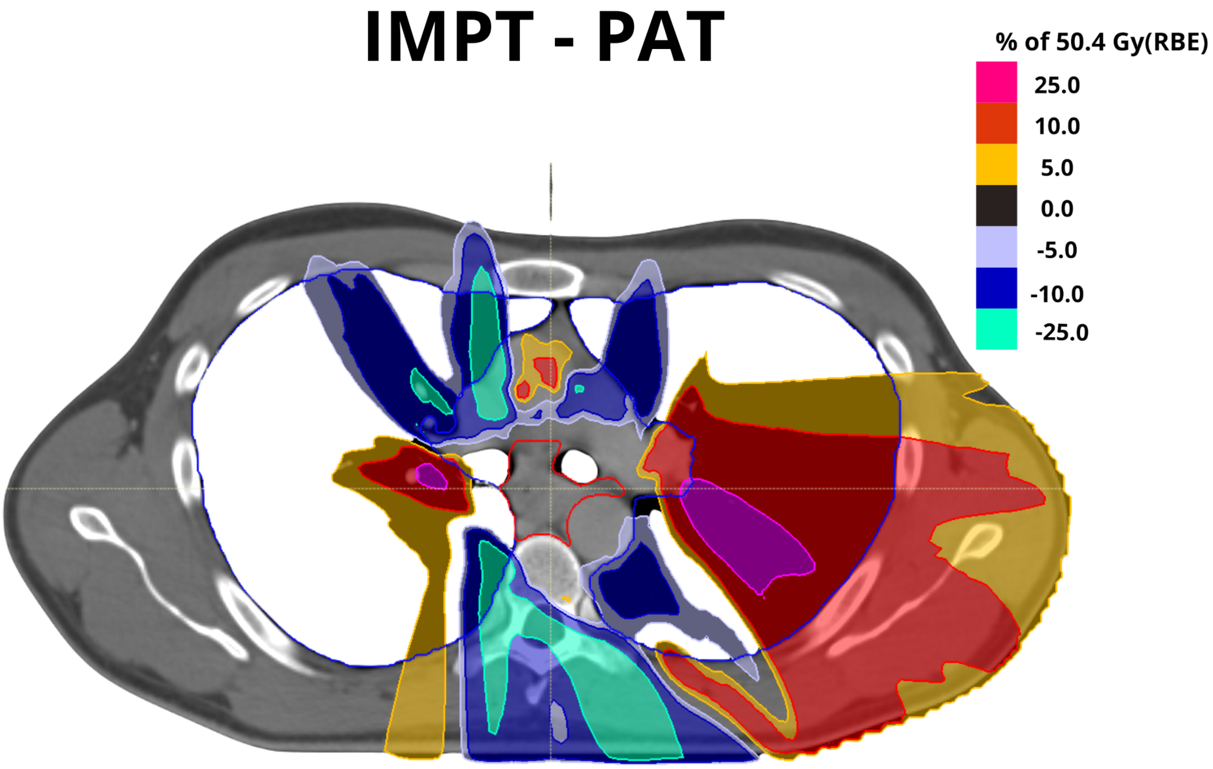

(b)

Figure S1: (a)Isodose comparison between IMPT and PAT for one patient (b)Dose map difference between techniques. It can be observed that PAT contributes to more low dose spreading within the body (10–25% increase).

Table S4: NTCP results for pericardial effusion and pneumonitis. Δ = IMPT - PAT > 0 means PAT is a superior technique for that metric

Strategy	NTCP pericardial effusion	NTCP pneumonitis
IMPT	12.9	1.4
PAT	12.3	1.4

(a) Median NTCP values.

NTCP metric	Δ	p
Pericardial effusion	0.5	$\ll 0.001$
Pneumonitis	0.1	0.001

(b) Median NTCP differences.

	n beams		Total EL		Total ELS up		Total ELS down		Total spots		BDT [s]		Travel time [s]	
Patient	IMPT	PAT	IMPT	PAT	IMPT	PAT	IMPT	PAT	IMPT	PAT	IMPT	PAT	IMPT	PAT
1	2	2	50	122	1	13	48	108	8377	18801	111.5	265.6	49.1	0
2	2	2	69	122	1	6	67	115	20171	38671	165.4	267.3	49.1	0
3	2	2	72	122	1	7	70	114	20091	43733	169.2	280.9	49.1	0
4	2	2	83	122	1	8	81	113	27928	90282	204.6	430.0	49.1	0
5	2	2	73	122	1	7	71	114	22956	35569	179.7	267.1	49.1	0
6	2	2	74	242	1	13	72	228	19176	34338	166.1	429.8	49.1	0
7	2	2	59	122	1	6	57	115	17043	41209	149.4	261.4	49.1	0
8	2	2	68	122	1	7	66	114	29083	48855	199.9	302.4	49.1	0
9	2	2	60	242	1	10	58	231	28904	71323	190.6	494.4	49.1	0
10	2	2	73	122	1	6	71	115	24591	62086	180.4	330.7	49.1	0
11	2	2	80	242	1	11	78	230	21033	46893	176.4	443.7	49.1	0
12	2	2	74	242	1	13	72	228	30710	84105	208.9	559.5	49.1	0
13	2	2	68	122	1	7	66	114	15050	70427	149.7	353.7	49.1	0
14	2	2	70	242	1	9	68	232	18778	57071	165.7	454.6	49.1	0
15	2	2	57	122	1	7	55	114	18911	22863	154.9	235.1	49.1	0
16	2	2	60	182	1	10	58	171	22462	50237	168.9	386.2	49.1	0
17	2	2	60	362	1	11	58	350	18867	51619	154.8	587.8	49.1	0

Table S5: Plan parameters, per patient and modality,affecting the BDT calculation.

References

[1] Populaire, P., Marini, B., Poels, K., Svensson, S., Sterpin, E., Fredriksson, A., et al. Autodelineation methods in a simulated fully automated proton therapy workflow for esophageal cancer. *Phys. Imaging Radiat. Oncol.* 2024; 32:100646. https://doi.org/https://doi.org/10.1016/j.phro.2024.100646.

[2] Paganetti, H. Range uncertainties in proton therapy and the role of Monte Carlo simulations. *Phys. Med. Biol.* 2012; 57:R99. https://doi.org/https://dx.doi.org/10.1088/0031-9155/57/11/R99.

[3] Engwall, E., Battinelli, C., Wase, V., Marthin, O., Glimelius, L., Bokrantz, R., et al. Fast robust optimization of proton PBS arc therapy plans using early energy layer selection and spot assignment. *Phys Med Biol* 2022; 67:065010. https://doi.org/10.1088/1361-6560/ac55a6.

[4] Kutcher, G. J. and Burman, C. Calculation of complication probability factors for non-uniform normal tissue irradiation: The effective volume method gerald. *Int. J. Radiat. Oncol. Biol. Phys.* 1989; 16:1623–1630. https://doi.org/https://doi.org/10.1016/0360-3016(89)90972-3.

[5] Semenenko, V. A. and Li, X. A. Lyman–Kutcher–Burman NTCP model parameters for radiation pneumonitis and xerostomia based on combined analysis of published clinical data. *Phys. Med. Biol.* 2008; 53:737. https://doi.org/https://doi.org/10.1088/0031-9155/53/3/014.

[6] Fukada, J., Fukata, K., Koike, N., Kota, R., and Shigematsu, N. Mean heart dose-based normal tissue complication probability model for pericardial effusion: a study in oesophageal cancer patients. *Sci. Rep.* 2021; 11:18166. https://doi.org/https://doi.org/10.1038/s41598-021-97605-9.
